# Successful Coronary Stent Retrieval from the Saphenous Vein Graft to Right Coronary Artery

**DOI:** 10.1155/2009/718685

**Published:** 2009-11-08

**Authors:** Mustafa Aydin, Muhammet Rasit Sayin

**Affiliations:** Department of Cardiology, School of Medicine, Zonguldak Karaelmas University, 67600, Kozlu Zonguldak, Turkey

## Abstract

Stent dislodgement and migration is a rare but serious complication of stent usage. For extraction of unexpanded stents different techniques have been described previously. We describe a case which used small baloon catheter for retrieval of a stent from the SVG-RCA.

## 1. Introduction

The utilization of coronary stents has been the most important advancement in the percutaneous treatment of coronary artery disease (CAD). A rare but potentially serious complication of stent usage is dislodgment of the stent from the balloon catheter before the deployment and subsequent dislocation of the stent into the vascular system. It may embolise in the coronary circulation and evoke cardiac infarction, which is an indication of emergency coronary artery bypass grafting (CABG).Alternatively, it may cause embolic cerebrovascular events, peripheral embolisation, or even death [1, 2].

For extraction of unexpanded stents from the coronary circulation, different percutaneous techniques have been described, including the use of balloon catheters, loop snares, two twisted guide wires, or retrieval devices (biliary or myocardial biopsy forceps, multipurpose baskets) [2].

## 2. Case Report

**Figure 1:**
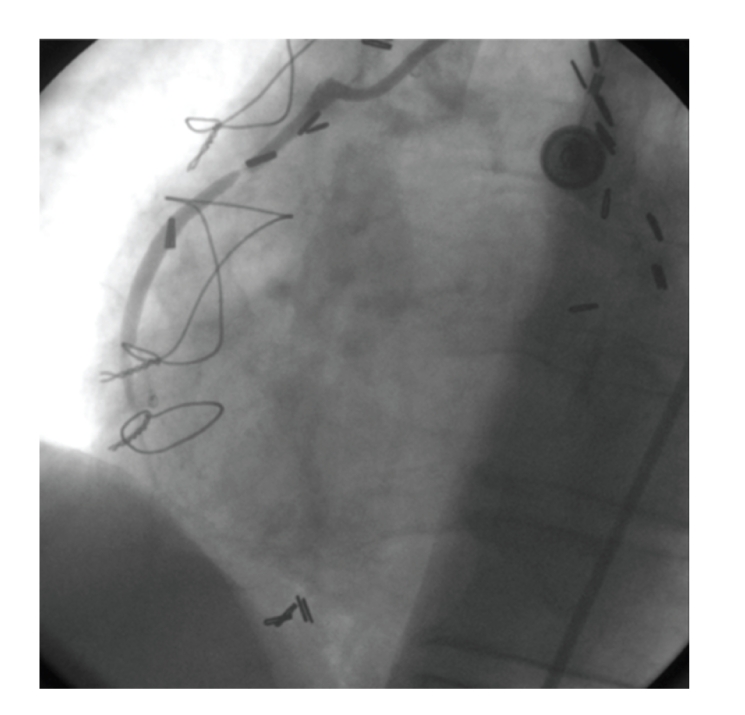
Saphen vein arteriogram showing 95% stenosis.

A 67-year-old male patient was admitted to hospital because of unstable angina. He had coronary artery bypass grafting (CABG) (internal mammary to left anterior descending artery, saphenous vein graft (SVG) to left circumflex artery, and SVG to right coronary artery (RCA)) seventeen years ago. Diagnostic catheterization (Figure 1) demonstrated 95% stenosis at the proximal of the SVG-RCA.

**Figure 2:**
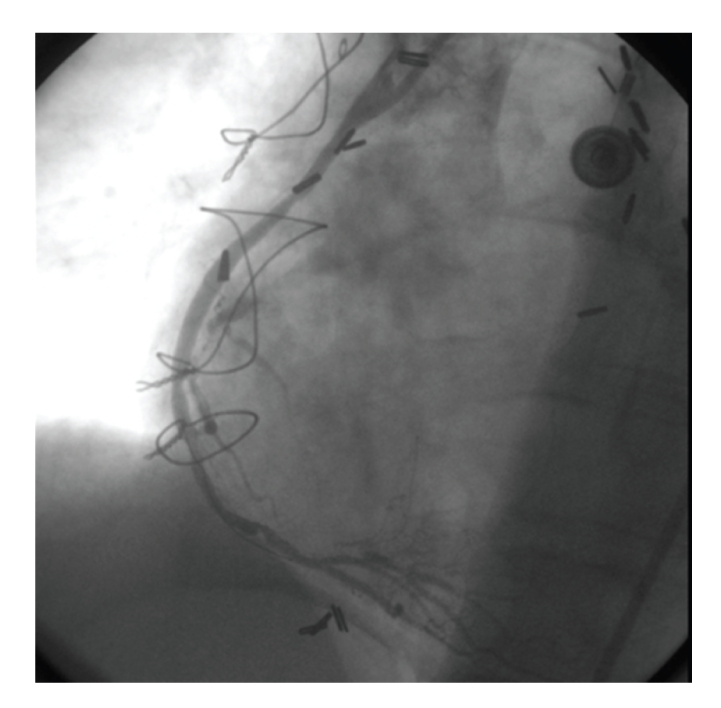
After balloon dilatation.

**Figure 3:**
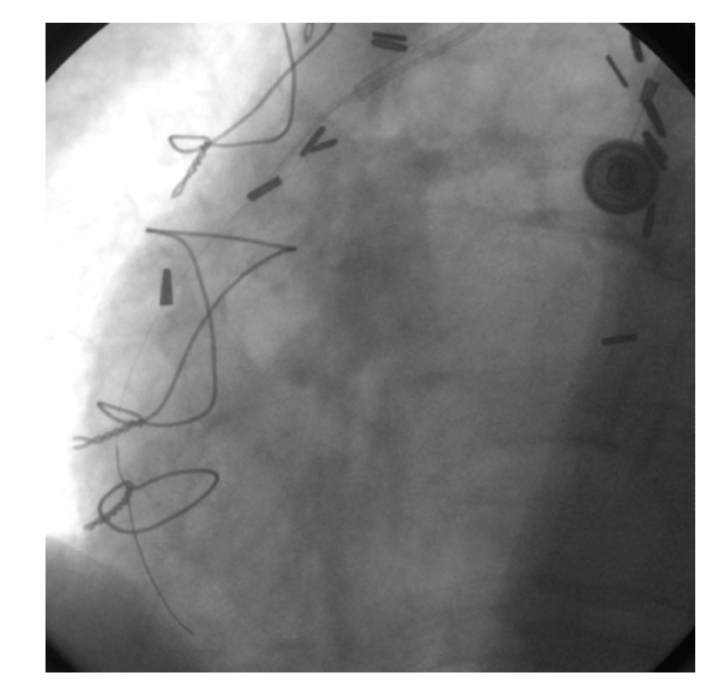
Stent had dislodged from the balloon catheter.

**Figure 4:**
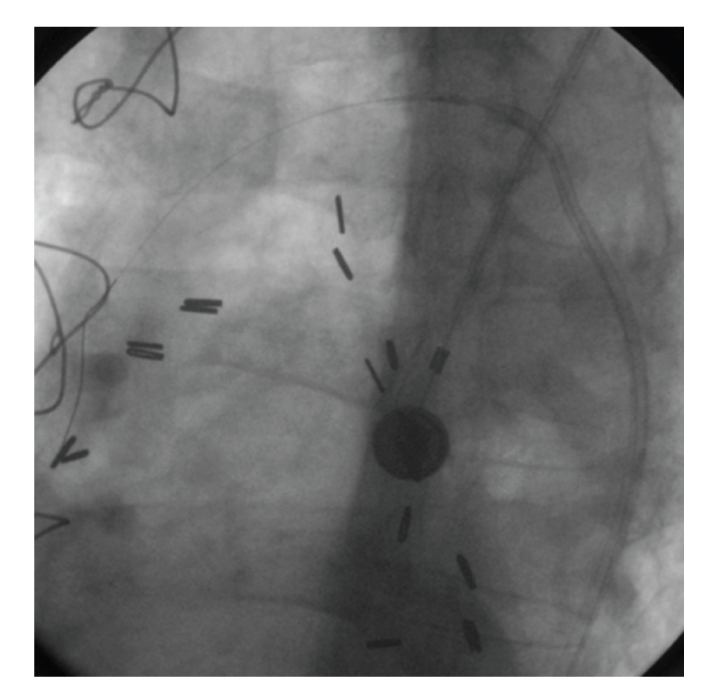
Small balloon, stent, and catheter complex.

The patient was referred for PCI. The target lesion was predilated with a 2.75 × 20 mm Asahi Intecc balloon. Subsequent angiography revealed 20% stenosis at the lesion site (Figure 2). The decision was made to place a stent due to the existing plaque fracture and the improved long-term outcome results already achieved with stents. A 3.0 × 18 mm Ephesos stent was selected. Stent could not be advanced through the lesion. After several attempts the stent was pulled back into the guiding catheter. During the withdrawing process the stent had dislodged from the balloon catheter (Figure 3). A small balon (1.25 × 10 mm Invatec Avion Plus) was advanced through the stent, inflated, and finally was withdrawn with the stent (Figure 4).

## 3. Discussion

Stent dislodgment and migration is an uncommon problem. But as a result of the universal use of premounted stents invasive cardialogists may come across with it more often. Stent dislodgement from the delivery system most often occurs while the stent-balloon assembly is pulled back into the guiding catheter, because the target lesion either could not be reached or could not be passed. Factors predisposing to the inability of stent delivery are poor support of the guiding catheter or the guide wire, vessel tortuosity proximal to the lesion, and severe vessel calcification [1–3]. In the event of stent dislodgment the primary objective should be the retrieval of dislodged stent from the coronary circulation due to the possibility of significant complications. For extraction of unexpanded stents from the coronary circulation different percutaneous techniques have been described, including the use of balloon catheters, loop snares, two twisted guide wires, or retrieval devices (biliary or myocardial biopsy forceps, multipurpose baskets) [2]. The use of small balloon catheters is effective, especially in cases where a stent is still riding on a guide wire and is deployed enough to advance a smallballoon catheter through its lumen [1, 2]. In our case the small balloon catheter was the right tool for successful retrieval of a stent from the SVG-RCA. The choice of retrieval technique should be specific to each case and to the operator's experience.
